# Human Double-Negative Regulatory T-Cells Induce a Metabolic and Functional Switch in Effector T-Cells by Suppressing mTOR Activity

**DOI:** 10.3389/fimmu.2019.00883

**Published:** 2019-04-26

**Authors:** Tabea Haug, Michael Aigner, Moritz M. Peuser, Carolin D. Strobl, Kai Hildner, Dimitrios Mougiakakos, Heiko Bruns, Andreas Mackensen, Simon Völkl

**Affiliations:** ^1^Department of Internal Medicine 5, Hematology and Oncology, University Hospital Erlangen, University of Erlangen-Nuremberg, Erlangen, Germany; ^2^Department of Internal Medicine 1, University Hospital Erlangen, University of Erlangen-Nuremberg, Erlangen, Germany

**Keywords:** double-negative T-cells, immune tolerance, mTOR, T-cell metabolism, allogeneic hematopoietic stem cell transplantation, GvHD

## Abstract

The recently discovered population of TCRαβ+ CD4–/CD8– (double-negative, DN) T-cells are highly potent suppressor cells in mice and humans. In preclinical transplantation models, adoptive transfer of DN T-cells specifically inhibits alloreactive T-cells and prevents transplant rejection or graft-vs.-host disease (GvHD). Interestingly, clinical studies in patients who underwent allogeneic stem cell transplantation reveal an inverse correlation between the frequency of circulating DN T-cells and the severity of GvHD, suggesting a therapeutic potential of human DN T-cells. However, their exact mode of action has not been elucidated yet. Investigating the impact of DN T-cells on conventional T-cells, we found that human DN T-cells selectively inhibit mTOR signaling in CD4 T-cells. Given that mTOR is a critical regulator of cellular metabolism, we further determined the impact of DN T-cells on the metabolic framework of T-cells. Intriguingly, DN T-cells diminished expression of glucose transporters and glucose uptake, whereas fatty acid uptake was not modified, indicating that DN T-cells prevent metabolic adaptation of CD4 T-cells upon activation (i.e., glycolytic switch) thereby contributing to their suppression. Further analyses demonstrated that CD4 T-cells also do not upregulate homing receptors associated with inflammatory processes. In contrast, expression of central memory-cell associated cell surface markers and transcription factors were increased by DN T-cells. Moreover, CD4 T-cells failed to produce inflammatory cytokines after co-culture with DN T-cells, whereas IL-2 secretion was enhanced. Taken together DN T-cells impair metabolic reprogramming of conventional CD4 T-cells by abrogating mTOR signaling, thereby modulating CD4 T-cell functionality. These results uncover a new mechanism of DN T-cell-mediated suppression, pointing out that DN T-cells could serve as cell-based therapy to limit alloreactive immune response.

## Introduction

Allogenic hematopoietic stem cell transplantation (allo-HSCT) is often the only curative treatment option for patients with leukemia, lymphoma, and other malignancies of the hematopoietic system (1). Despite advances in allo-HSCT (2), life-threatening treatment-related complications can arise amongst others because donor T-cells recognize not only the recipient's malignant tumor cells (graft-vs.-tumor effect, GvT), but also target healthy tissue of transplanted recipients (graft-vs.-host disease, GvHD) (3). Standard therapy of GvHD with corticosteroids is insufficient as 50% of the patients are steroid-refractory and systemic immunosuppression carries the risk of cancer relapse and opportunistic infections (4). Alternative treatment strategies to specifically inhibit or modulate alloreactive T-cells could improve the outcome and survival rate of allo-HSCT (5). One promising approach to limit exaggerating T-cell responses could be the use of regulatory T-cells (Treg) as an adoptive cellular therapy. In first clinical trials, infusion of *ex vivo* expanded Tregs was reported to be safe, feasible, and capable of reducing GvHD after allo-HSCT (6, 7).

In fact, T-cell receptor (TCR) αβ+ CD4–/CD8– double-negative regulatory (DN) T-cells compose 1–5% of all T-cells in mice and humans and display immunoregulatory functions with therapeutic potential *in vitro* and *in vivo* (8–10). Notably, murine DN T-cells have been shown to suppress auto-, allo-, and xenogenic immune responses in a broad spectrum of murine disease models (11–15). Accordingly, adoptive transfer of DN T-cells prevented rejection of major histocompatibility complex (MHC–) mismatched organ transplants (10, 16) or the onset of diabetes (17). In particular, the transfer of murine DN T-cells after allo-HSCT resulted in induction of tolerance in allogenic T-cells, thereby avoiding GvHD while maintaining anti-leukemia effects (18). Moreover, clinical relevance for human DN T-cells was revealed since frequency of circulating DN T-cells in patients undergoing allo-HSCT is inversely correlated with the severity of acute GvHD (19). The observation that patients with frequencies of DN T-cells over 1% did not develop any severe acute GvHD favors these cells as a promising tool for cellular therapy. In addition, a recent report disclosed DN T-cell numbers to be lowered in patients at the point of chronic GvHD commencement (20). Of interest, human DN T-cells were also shown to delay the onset of xenogeneic GvHD in a humanized mouse model (21). Murine DN T cells have been reported to mediate immune suppression via Fas-FasL interactions, secretion of perforin/granzyme or indirectly via modification of dendritic cells (DCs) (11, 13, 14, 22). However, human DN T-cells do not eliminate responder cells, modulate DCs or deplete nutrients or T-cell growth factors. Although TCR activation, cell-cell-contact, and *de-novo* protein synthesis were essential for human DN T cell-mediated suppression (9), the manner in which DN T-cells shape reactive T-cells has not been defined.

In order to understand the impact of DN T-cells on alloreactive T-cells, we investigated the fate and function of DN T-cell-treated CD4 T-cells. We found that DN T-cells suppress proliferation, but also modify metabolism, characteristics, and effector functions of CD4 T-cells by selective blocking of the mTOR (mammalian target of rapamycin) signaling pathway. Taken together these results suggest that DN T-cells might bias CD4 T-cells toward a quiescent phenotype thereby inducing peripheral tolerance after allo-HSCT.

## Materials and Methods

### Medium and Reagents

T-cells were cultured in RPMI 1640 medium supplemented with 10% human AB-serum (c.c.pro, Oberdorla, Germany). The following recombinant human cytokines were used: 100 U/ml IL-2 (Novartis, Basel, Switzerland), 500 U/ml granulocyte-macrophage colony-stimulating factor (GM-CSF) (Sanofi, Paris, France), 5 ng/ml IL-4 and transforming growth factor beta (TGF-β) (PeproTech, Hamburg, Germany), 10 ng/ml IL-1β and tumor necrosis factor (TNF) (PromoKine, Heidelberg, Germany), 1,000 U/ml IL-6 (CellGenix, Freiburg, Germany), and 1 μg/ml prostaglandin E2 (PGE2) (Enzo Life Science, Lörrach, Germany).

### Isolation and Culture of T-Cells

Peripheral blood mononuclear cells (PBMCs) were separated by density gradient centrifugation from leukapheresis products from healthy volunteers using Pancoll (PAN Biotech, Aidenbach, Germany). The study was approved by the Ethics committee of the University Erlangen-Nuremberg (protocol number 284_18 Bc). Informed consent was provided in accordance with the Declaration of Helsinki. Isolation of CD4 T-cells (human CD4+ T cell isolation kit) and DN T-cells (human double-negative T cell isolation kit) from PBMCs via magnetic separation was performed according to the manufacturer's instructions (Miltenyi Biotec, Bergisch-Gladbach, Germany). DCs were generated as previously described (23). In brief, monocytes were enriched by adherence to plastic surface of cell culture flasks for 2 h, then cultured with medium plus 10% fetal calf serum (FCS) supplemented with GM-CSF, IL-4, and TGFβ. On day 5, GM-CSF, IL-4, TNF, IL-6, IL-1β, and PGE2 were added to the culture for an additional 48 h, non-adherent cells were harvested and used for stimulation of DN T-cell cultures. DN T-cells (1 × 10^5^/well) from donor A were co-cultured with allogeneic mature DCs (2.5 × 10^4^/well) from donor B in 96-well plates in complete medium plus IL-2 (100 IU/ml). DN T-cells were re-stimulated weekly with allogeneic DCs for 2–5 weeks. Viability and purity of the T-cells was monitored by flow cytometry. Further purification via magnetic bead separation was performed if purity was <95%. DN T-cells were used for functional assays not earlier than 5 days after the last stimulation.

### T-Cell Suppression Assays

Freshly isolated CD4 T-cells from donor A were labeled with a violet proliferation dye (VPD450 BD Biosciences, Heidelberg, Germany) and seeded in 96-well plates (5 × 10^4^ CD4 T-cells per well). DN T-cells from donor A were used as suppressor cells in a responder to suppressor ratio of 1:1. Cells were activated with anti-CD3/CD28 coated Dynabeads (ThermoFisher, Waltham, USA) at a ratio of 25:1. After 1, 3, or 6 days of co-culture, cells were harvested, stained with monoclonal antibodies (mAbs) or dyes, and measured by flow cytometry. CD4 T-cells were analyzed by gating on viable lymphocytes with forward scatter-area (FSC-A)/sideward scatter-area (SSC-A) and on singlets with forward scatter-height (FSC-H), followed by gating on CD4+ cells. The proliferation of CD4 responder T-cells was analyzed by the decrease in proliferation dye fluorescence. Unstimulated CD4 T-cells were used as a control. DN T-cells used in suppression assays for cytokine detection were additionally labeled with carboxyfluorescein succinimidyl ester (CFSE) (Sigma, Munich, Germany). For activation experiments, CD4 T-cells were labeled with VPD450 and incubated with the Akt/mTOR signaling activator SC79 (20 μg/ml, Calbiochem/Merck, Darmstadt, Germany), the mTOR signaling activator MHY-1485 (20 μM, Selleckchem, Munich, Germany) or medium only as a control. After 2 h, cells were washed twice and used as responder cells in a co-culture as described above. To analyze the impact of mTOR hyperactivation using MHY-1485 on DN T-cell induced alterations, cells were harvested at day 3 of co-culture and their phenotype and metabolism was assessed by flow cytometry. Proliferation of T-cells was determined by flow cytometry at day 6 of co-culture.

### Transwell Assays

For transwell experiments, CD4 T-cells were activated with anti-CD3/CD28 coated beads in the bottom of a 24-well plate at a 25:1 ratio. In total 5 × 10^5^ were seeded per well. DN T-cells were added to the bottom well or together with anti-CD3/CD28 coated beads to the top chamber (Corning, New York, USA). Top and bottom chamber were separated by a 0.4 μm permeable pore polycarbonate membrane that allows pass through of soluble factors, but not of cells. Controls were cultured in a 24-well plate as described above.

### Flow Cytometry

Cells were stained with anti-human anti-CD4 (SK3), anti-CD27 (L128), anti-pS6 (pS240 N4-41), anti-p-p38 (p180/pY182), anti-CD95 (DX2), anti-CD98 (UM7F8), anti-IFN-γ (B27), anti-IL-2 (MQ1-17H12), anti-IL17-A (N49-653), anti-CD195 (2D7), anti-CCR5 (J252D4), anti-CD54 (HA58), anti-NF-κB p65 [(pS529) (K10-895.12.50)] (all from BD Biosciences, Heidelberg, Germany), anti-T-bet (eBio4510), anti-CD28 (10F3), anti-Eomes (WG1928, all from ThermoFisher, Waltham, USA), anti-CD197 (G043H7), anti-HIF-1α (546-16), anti-CD183 (G025H7), anti-GM-CSF (BV D2-21C11), anti-CD49d (9F10), and anti-Integrin β7 (FIB504) (all from Biolegend, San Diego, USA), anti-GLUT1 (EPR3915) and anti-GLUT3 (polyclonal, both from Abcam, Cambrige, United Kingdom) mAbs. Intracellular glucose transporter 1 (GLUT1), GLUT3, HIF-1α, interferon-gamma (IFN-γ), IL-2, GM-CSF, IL-17A were stained with the Cytofix/Cytoperm kit (BD Biosciences, Heidelberg, Germany), according to the manufacturer's protocol. Intracellular T-box transcription factor (T-bet) and eomesodermin (Eomes) were stained with Foxp3 Transcription Factor Staining Buffer Set (ThermoFisher, Waltham, USA), according to the manufacturer's protocol. Lymphocytes were determined by FSC-A/SSC-A, doublets were excluded by FSC-H and CD4+ cells were gated by the indicated mAbs. For detection of phosphorylated proteins on day 1 after co-culture, cells were stained with surface mAbs, washed, fixed with BD Cytofix/Cytoperm, washed again, and permeabilized with Perm Buffer III (BD Biosciences, Heidelberg, Germany) for 30 min. After washing with PBS (2% FCS), cells were incubated with the indicated mAbs for 40 min, washed, and fixed with CellFIX (BD Biosciences, Heidelberg, Germany). For assessment of glucose or fatty acid uptake cells were incubated with 2-(N-(7-Nitrobenz-2-oxa-1,3-diazol-4-yl)Amino)-2-Deoxyglucose (2-NBDG) (200 μM, AAT Bioquest, Sunnyvale, California, USA) for 10 min or Bodipy_C1−12_ (2 μM, life technologies, Carlsbad, California, USA) for 5 min at 37°C and washed twice with PBS (2% FCS). Flow cytometry data were acquired on a FACS Canto II (BD Biosciences, Heidelberg, Germany) and CD4 T-cells were analyzed for indicated mAbs with FlowJo software (TreeStar, Ashland, Oregon, USA).

### Detection of Cytokines

To ascertain cytokines in CD4 T-cells, co-cultures were treated with phorbol-12-myristat-13-acetat (PMA) (2 μg/ml)/Ionomycin (1 μM, both Sigma-Aldrich, Munich, Germany) for 4 h on day 6 of co-culture. Intracellular staining was performed as indicated above. For measurement of secreted cytokines on day 6 of co-culture, DN T-cells and CD4 T-cells were separated using anti-CD4+ magnetic beads (Miltenyi Biotec, Bergisch-Gladbach, Germany). Purity was confirmed with flow cytometry (>95%). CD4 T-cells were stimulated with PMA/Ionomycin, supernatants were collected after 6 h and analyzed simultaneously for IL-2, IL-4, IL-6, IL-10, IL-17A, TNF, and IFN-γ secretion using the human Th1/Th2/Th17 cytokine cytometric bead array kit (BD Biosciences, Heidelberg, Germany).

### T-Cell Migration Assay

To measure the migration of T-cells, CD4 T-cells were incubated unstimulated, with anti-CD3/CD28 beads or with anti-CD3/CD28 beads and DN T-cells as described above. On day 6 of co-culture CD4 T-cells and DN T-cells were separated using anti-CD4+ magnetic beads (Miltenyi Biotec, Bergisch-Gladbach, Germany). Purity was confirmed with flow cytometry (>95%). CD4 T-cells (10^5^) were re-suspended in RPMI medium without human AB serum and deposited on the upper chamber of a transwell insert (5.0 μm pore size, Corning Inc., New York, USA). The bottom well contained RPMI medium only or with 100 ng/ml CXCL10, CCL3, or CXCL9 (Biolegend, San Diego, California, USA). Transwell plates were incubated for 2 h at 37°C. The content of the lower chamber was collected, stained with anti-human anti-CD4, and migrated cell numbers were quantified by usage of 123 counting Beads (Thermo Fisher, Waltham, USA).

### ELISA

For measurement of Akt and p38 phosphorylation with Fast Activated Cell-based ELISA (FACE), CD4 T-cells were activated with anti-CD3/CD28 coated beads in the presence or absence of DN T-cells. After 24 h, CD4 T-cells were separated using anti-CD4+ magnetic beads (Miltenyi Biotec, Bergisch-Gladbach, Germany). Purity was confirmed with flow cytometry (>95%). CD4 T-cells (10^4^) were plated on a Poly-D-Lysin coated (10 mg/ml) 96-well flat bottom plate and fixed with Formaldehyde. For detection of phosphorylation, we used the FACE p38 in-cell Western analysis for phospho-p38 (T180/Y182) and FACE AKT in-cell Western analysis for phospho-AKT (S473) (Active motif, Carlsbad, California, USA) according to the manufacturer's instructions.

### Metabolic Flux Analyses

The CD4 T-cells' bioenergetics after DN T-cell co-cultured was assessed using an XFe96 Extracellular Flux Analyzer (Seahorse Bioscience, North Billerica, Massachusetts, USA). CD4 T-cells were activated with anti-CD3/CD28 coated beads in the presence or absence of DN T-cells, unstimulated CD4 T-cells were used as control. On day 3 of co-culture CD4 T-cells and DN T-cells were separated using anti-CD4+ magnetic beads (Miltenyi Biotec, Bergisch-Gladbach, Germany). Purity and viability was confirmed with flow cytometry (>95%). To determine oxygen consumption rate (OCR) and extracellular acidification rate (ECAR) CD4 T-cells were utilized for XF Mitochondrial Stress Test Kits and XF Glycolysis Stress Test Kits according to the manufacturer's recommendations (Seahorse Bioscience, North Billerica, USA) and as previously detailed (24, 25).

### Statistical Analyses

Data were analyzed with Graphpad Prism software (GraphPad San Diego, USA). Results were compared using non-parametric (Mann-Whitney-U or Wilcoxon) tests. A *p* < 0.05 was considered significant.

## Results

### DN T-Cells Modulate TCR Signaling in CD4 T-Cells

**Figure 1:**
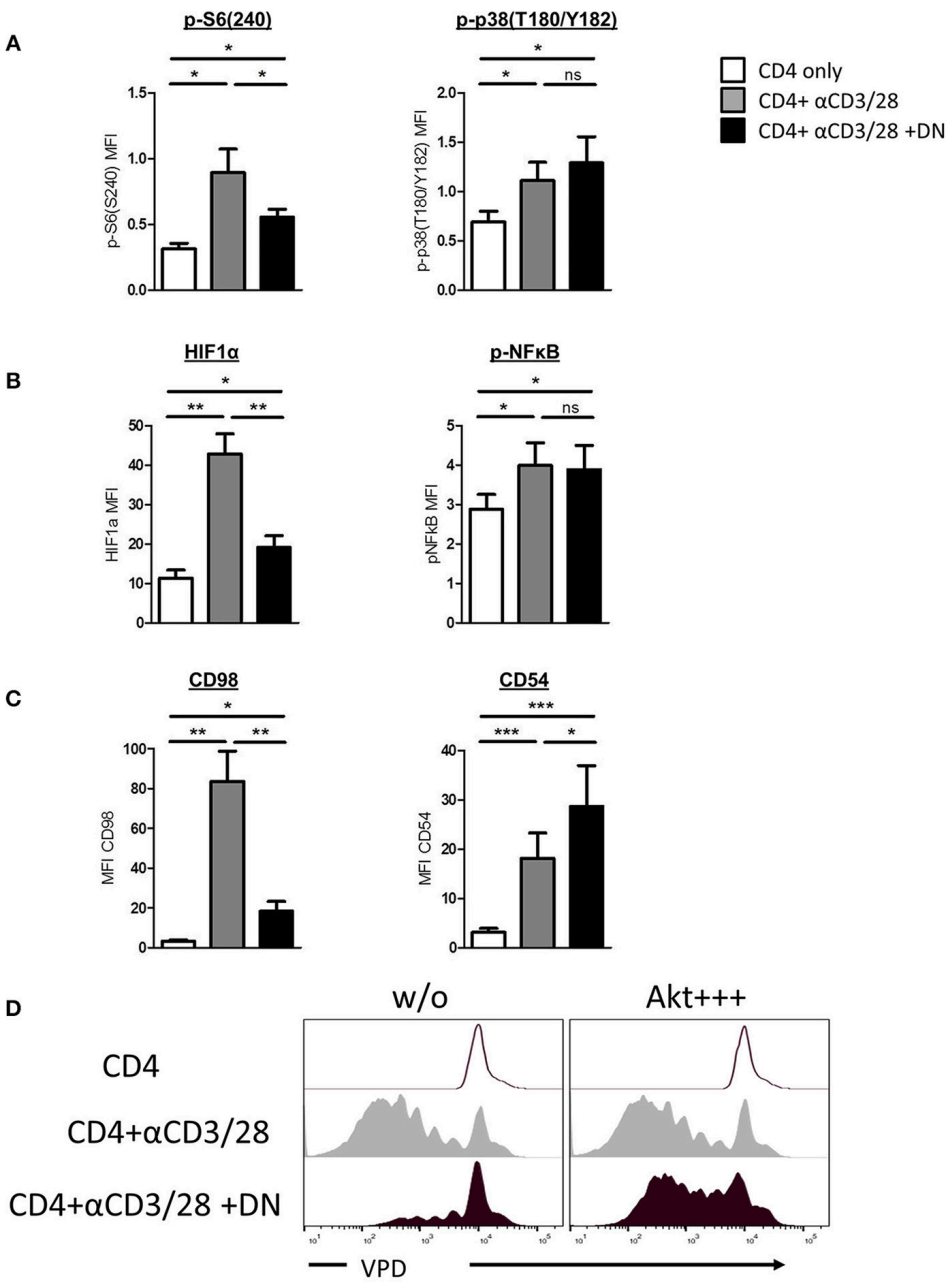
DN T-cells inhibit mTOR activation but not MAPK p38 signaling in CD4 T-cells. Freshly isolated CD4 T-cells were incubated with anti-CD3/CD28 coated beads in absence (gray) or presence (black) of DN T-cells. Unstimulated CD4 T-cells were used as negative control (white). **(A)** Phosphorylation of ribosomal protein S6(S240) (left) and MAPK p38(T180/Y182) (right) in CD4 T-cells after 24 h culture was quantified by flow cytometry. Graphs show MFI +/-SEM of at least six independent experiments. **(B)** Expression of HIF-1α and NFκB(p65) was analyzed in CD4 T-cells after 24 h co-culture, graph represent MFI +/–SEM of 7 experiments. **(C)** Expression of CD98 and CD54 was measured after 3 days, MFI +/SEM of at least five experiments is shown. Ns, not significant, ^*^p < 0.05, ^**^p < 0.01, ^***^p < 0.01. **(D)** Freshly isolated VPD-labeled CD4 T-cells were incubated with SC79 (Akt+ + +) for 2 h at 37°C and washed intensively. Treated and untreated VPD-labeled CD4 T-cells were activated with anti-CD3/CD28 coated beads in presence or absence of DNT-cells for 6 days. Cells were analyzed by flow cytometry, histograms were gated for CD4 T-cells.

Human DN T-cells effectively inhibit CD4 T-cell proliferation but the consequences for suppressed CD4 T-cells remain elusive. Given that initial signal transduction after TCR ligation plays a pivotal role for the further fate of the cell, we first focused on whether human DN T-cells can influence CD4 T-cell signaling. We addressed this question by investigating phosphorylation of central signaling molecules in CD4 T-cells after stimulation with and without DN T-cells via flow cytometry. Activated CD4 T-cells exhibited high phosphorylation levels at the downstream molecule of the mTOR signaling pathway S6, while DN T-cell-treated CD4 T-cells revealed lower S6 phosphorylation (Figure 1A and Supplemental Figure 1A). However, DN T-cells did not influence the phosphorylation of the signaling molecule mitogen-activated protein (MAP) kinase p38, pointing to a selective modulation of signal transduction. To verify this finding, ELISA of total and phosphorylated Akt and p38 was performed. CD4 T-cells showed no differences of total-Akt expression in presence or absence of DN T-cells, while phospho-Akt was reduced in CD4 T-cells after co-culture with DN T-cells (Supplemental Figure 1B). In contrast, phosphorylation of p38 was not affected by DN T-cells. To further elucidate signaling alterations caused by DN T-cells, we analyzed if downstream targets of mTOR and p38 were also affected by DN T-cells. Notably, DN T-cells diminished the upregulation of mTOR-regulated transcription factor HIF-1α in CD4 T-cells, whereas activation of transcription factor NF-κB was not impaired (Figure 1B and Supplemental Figure 1C). Given that the cell surface molecule CD98 is upregulated due to mTOR activity (26) while CD54 is induced by p38 signaling (27), we analyzed the expression of these proteins as surrogate markers. CD98 but not CD54 expression was abrogated in activated CD4 T-cells after co-culture with DN T-cells (Figure 1C and Supplemental Figure 1D), underpinning that DN T-cells selectively inhibit mTOR signaling. To test the physiological relevance of mTOR signaling for DN T-cell-mediated suppression, we hyperactivated this pathway in CD4 T-cells using the small molecule SC79 as described in *Materials and Methods*. SC79 did not modify the proliferation of unstimulated and anti-CD3/CD28-coated beads activated CD4 T-cells. In contrast, pretreatment of CD4 T-cells with the mTOR-activator rendered the CD4 T-cells unsusceptible to DN T-cell-mediated suppression (Figure 1D). Taken together, these findings indicate that DN T-cells mediate their suppressive activity by decreasing mTOR activity rather than blocking entire signaling processes in CD4 T-cells.

### DN T-Cells Inhibit Glycolytic Reprogramming of CD4 T-Cells

**Figure 2:**
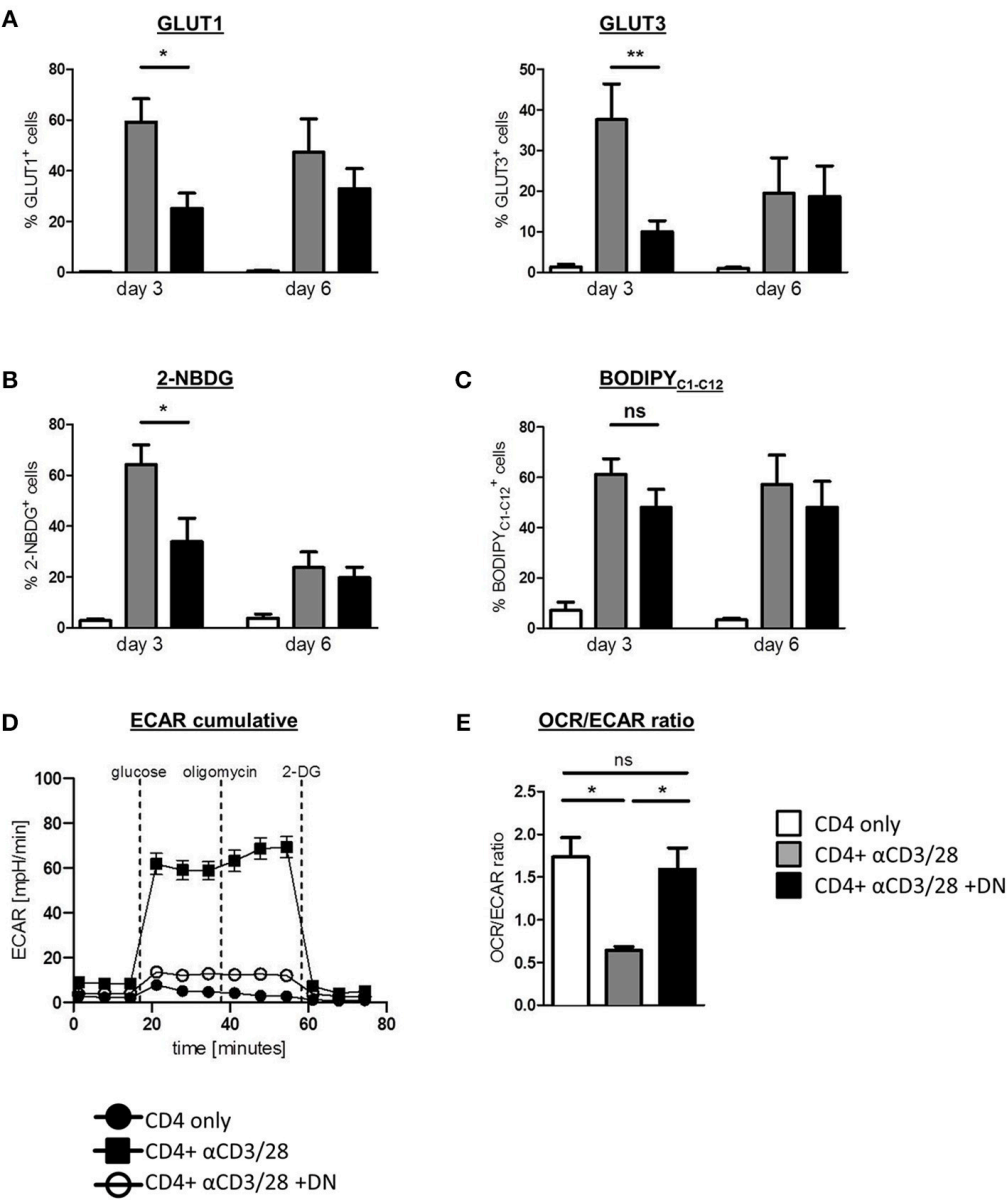
DN T-cells impair metabolic reprogramming of CD4 T-cells. Freshly isolated CD4 T-cells were incubated with anti-CD3/CD28 coated beads in absence (gray) or presence (black) of DN T-cells, unstimulated CD4 T-cells served as negative control (white). Cells were analyzed by flow cytometry after 3 and 6 days. **(A)** Expression of GLUT1 and GLUT3 in CD4 T-cells was determined by flow cytometry after 3 and 6 days. **(B)** Uptake of the glucose analog 2-NBDG and **(C)** the fatty acid Bodipy_C1−C12_ in CD4 T-cells was measured as described in *Materials and Methods*. Data of at least seven independent experiments +/- SEM are shown. **(D)** On day 3 of co-culture CD4 T-cells were re-isolated by magnetic sorting and ECAR was measured in CD4 T-cells, using an XFe96 flux analyzer. **(E)** The OCR/ECAR ratio indicative for the energetic balance between OXPHOS and aerobic glycolysis was calculated for unstimulated CD4 T-cells and for activated CD4 T-cells in presence or absence of DN T-cells (*n* = 4). ns, not significant, ^*^p < 0.05, ^**^p < 0.01.

Given that DN T-cells selectively inhibit the glycolytic key regulators mTOR and HIF-1α, we next considered whether DN T-cells affect CD4 T-cell metabolism. We therefore analyzed the regulation of glucose transporter 1 (GLUT1) and 3 (GLUT3) in the absence or presence of DN T-cells. Activation of CD4 T-cells with anti-CD3/CD28 coated beads induced high GLUT1 and GLUT3 expression that peaked after three days (Figure 2A and Supplemental Figure 2A). This upregulation of glucose transporters was impaired in the presence of DN T-cells, suggesting a reduced capacity for glycolysis. As a result of declined activation of CD4 T-cells after 6 days, differences between activated and DN T-cell-suppressed CD4 T-cells had vanished at that point. To verify the impact of mTOR signaling on DN T-cell-caused metabolic alterations, we activated this pathway using the specific activator MHY-1485 as described in *Materials and Methods*. MHY-1485 did not modify expression of GLUT1 in unstimulated and activated CD4 T-cells, while downregulation of GLUT1 expression was not present in mTOR hyperactivated CD4 T-cells in co-culture with DN T-cells (Supplemental Figure 2B). To further confirm the effect of DN T-cells on CD4 T-cell metabolism, we directly measured glucose and fatty acid uptake using the fluorescent analogs 2-NBDG and Bodipy_C1−12_. As expected, activation of CD4 T-cells with anti-CD3/CD28 coated beads resulted in high 2-NBDG consumption that was strongly impaired by DN T-cells (Figure 2B and Supplemental Figure 2C). In contrast, DN T-cells did not affect enhanced fatty acid uptake of CD4 T-cells (Figure 2C), demonstrating a selective inhibition of glucose utilization. In addition, real-time analyses of extracellular acidification rate (ECAR, indicative for aerobic glycolysis) as well as of oxygen consumption rate (OCR, indicative for mitochondrial respiration) of CD4 T-cells declared a reduced glycolytic rate upon incubation with DN T-cells (Figure 2D). When analyzing the OCR/ECAR ratio, an indicator for the balance between aerobe glycolysis and oxidative phosphorylation (OXPHOS), we noticed that the activation-related skewing of CD4 T-cells toward ECAR was abrogated by DN T-cells suggesting DN T-cells inhibited upregulation of glycolysis in activated CD4 T-cells (Figure 2D).

### DN T-Cells Affect the Phenotype of CD4 T-Cells

**Figure 3:**
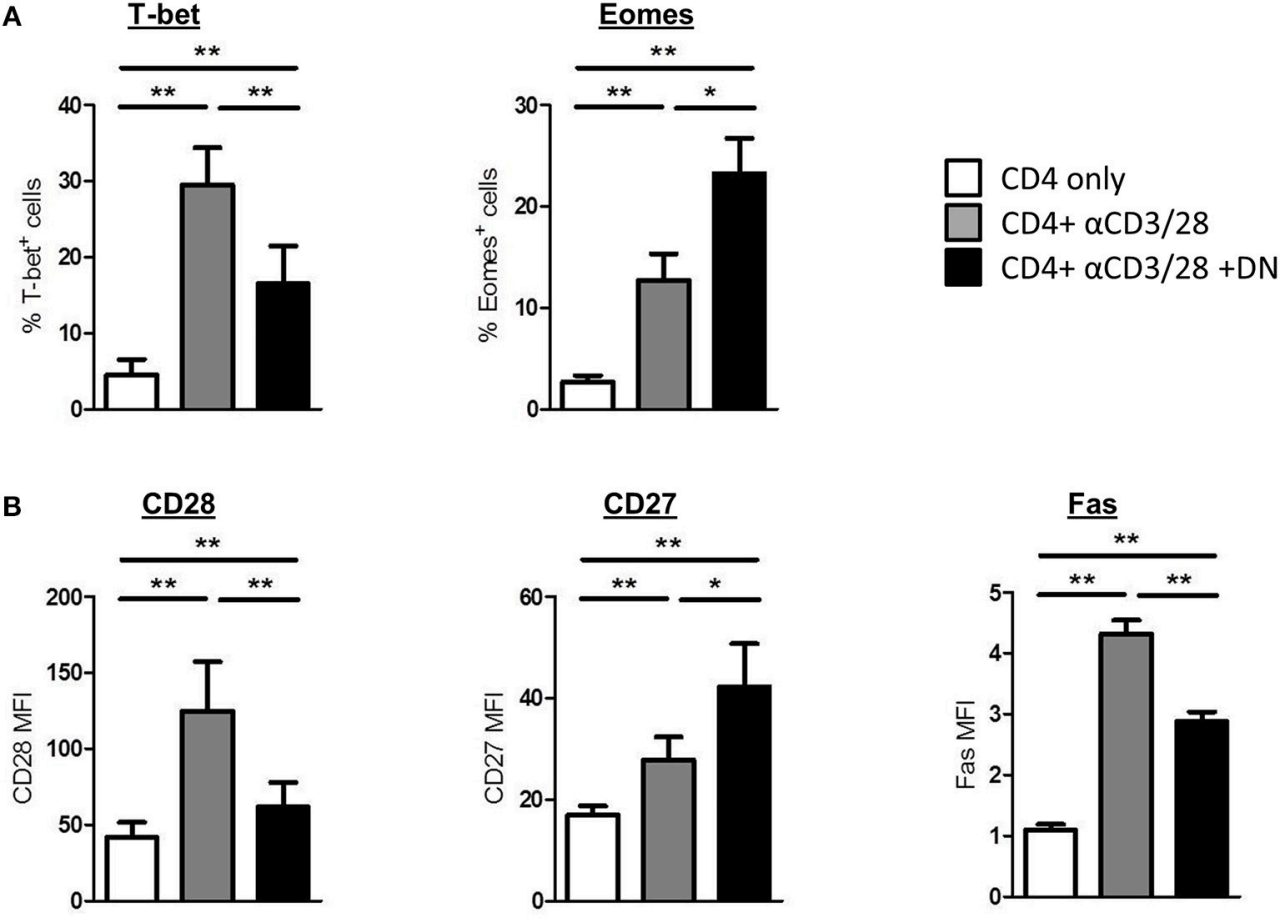
DN T-cells modulate expression profiles of CD4 T-cells. Freshly isolated CD4 T-cells were cultured with anti-CD3/CD28 coated beads in absence (gray) or presence (black) of DN T-cells, unstimulated CD4 T-cells were used as negative control (white). Cells were harvested on day 6 of co-culture and analyzed by flow cytometry. **(A)** CD4 T-cells were analyzed for the expression of transcription factors T-bet and Eomes. **(B)** Expression of CD28, CD27, and Fas on CD4 T-cells is shown. Data represent results of at least seven independent experiments. ^*^p < 0.05, ^**^p < 0.01.

To further explore the consequences of an altered metabolism in CD4 T-cells, we performed flow cytometry staining of intracellular and surface markers on day 6 of co-culture. First, we analyzed whether DN T-cells manipulate T-bet and Eomes expression in CD4 T-cells. Interestingly, DN T-cells diminished the induction of T-bet in activated CD4 T-cells, whereas Eomes expression was further enhanced after co-culture (Figure 3A and Supplemental Figure 3A). Furthermore, DN T-cells did not strengthen the expression of the transcription factor FoxP3 in CD4 T-cells (Supplemental Figure 3D). Since transcription factors orchestrate the expression of distinct T-cell markers, DN T-cells suppressed upregulation of the co-stimulatory cell surface molecule CD28 and the death receptor Fas on CD4 T-cells (Figure 3B and Supplemental Figure 3B). In contrast, expression of the co-stimulatory receptor CD27 was not reduced but further enhanced in presence of DN T-cells. To test the physiological relevance of mTOR signaling for DN T-cell-mediated characteristic changes, we overactivated mTOR in CD4 T-cells via MHY-1485. Notably, MHY-1485 treated activated CD4 T-cells were resistant to DN T-cell-induced downregulation of CD98 and CD28, whereas controls were not altered by mTOR hyperactivation (Supplemental Figure 3C). To further assess the differentiation of CD4 T-cells we stained for CCR7 and CD45RO (Supplemental Figure 3E). Unstimulated CD4 T-cells express CCR7 but are negative for CD45RO, while activated CD4 T-cells upregulate CD45RO and loose CCR7 on their surface. Of interest, DN T-cell co-cultured CD4 T-cells express CD45RO as well as CCR7. Overall, these results indicate that DN T-cells modulate the phenotype of suppressed CD4 T-cells.

### DN T-Cells Change Migratory Capacity of CD4 T-Cells

**Figure 4:**
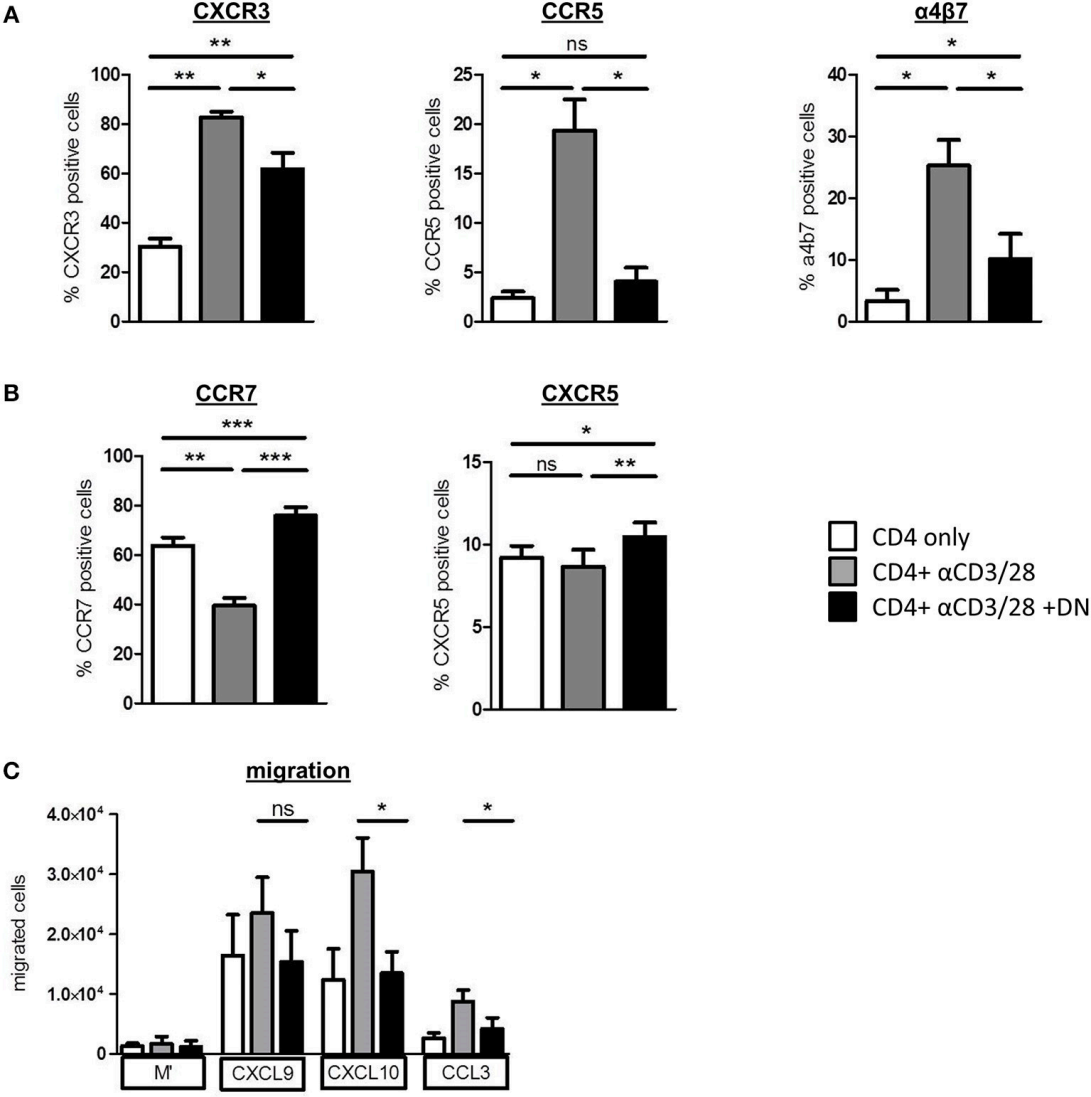
DN T-cells influence migratory capacity of CD4 T-cells. CD4 T-cells were incubated with anti-CD3/CD28 coated beads absence (gray) or presence (black) of DN T-cells, unstimulated CD4 T-cells were used as negative control (white). After 6 days, cells were analyzed by flow cytometry. Data represent expression of **(A)** CXCR3, CCR5, integrin a4b7, and **(B)** CCR7 and CXCR5 on CD4 T-cells. Mean percentages +/– SEM of seven independent experiments is shown. **(C)** On day 6 of co-culture CD4 T-cells were re-isolated by magnetic sorting and added to the upper chamber of an *in vitro* Transwell migration-assay with the indicated chemokines in the lower chamber. Chemokine dependent CD4 T cell migration was determined after 2 h with 123-counting beads by flow cytometry. ns, not significant, ^*^p < 0.05, ^**^p < 0.01, ^***^p < 0.001.

To investigate whether DN T-cells can restrict homing of alloreactive T-cells, we analyzed the expression of pro-inflammatory chemokine receptors CXCR3 and CCR5 that are associated with GvHD induction and severity (28, 29). Both CXCR3 and CCR5 and also the integrin α4β7, which is essential for homing of cells to the gut, were upregulated on CD4 T-cells after activation (Figure 4A and Supplemental Figure 4A). Of importance, DN T-cells diminished expression of GvHD-associated chemokine receptors, whereas CCR7 and CXCR5 were upregulated (Figure 4B and Supplemental Figure 4B), suggesting an augmented potential of CD4 T-cells to migrate to lymphoid organs. Moreover, mTOR hyperactivation with MHY-1485 reversed DN T-cell-related suppression of CXCR3 in CD4 T-cells (Supplemental Figure 4C), indicating that blocking expression of pro-inflammatory homing receptors was controlled by mTOR inhibition. To test the functional impact of differently expressed chemokine receptors after DN T-cell co-culture on the migratory capacity of CD4 T-cells, we performed an *in vitro* migration assay. CXCL9 and CXCL10 are ligands of CXCR3, while CCL3 binds to the CCR5 receptor. After activation CD4 T-cells migrated toward CXCL9, CXCL10, and CCL3 gradient (Figure 4C). However, migration of CD4 T-cells that were co-cultured with DN T-cells was declined pointing toward a limited ability of these cells to home to GvHD-target organs. These data extend our previous finding that DN T-cells modify CD4 T-cell expression profiles, thereby shaping their migratory patterns.

### DN T-Cells Modulate Effector Functions in CD4 T-Cells

**Figure 5:**
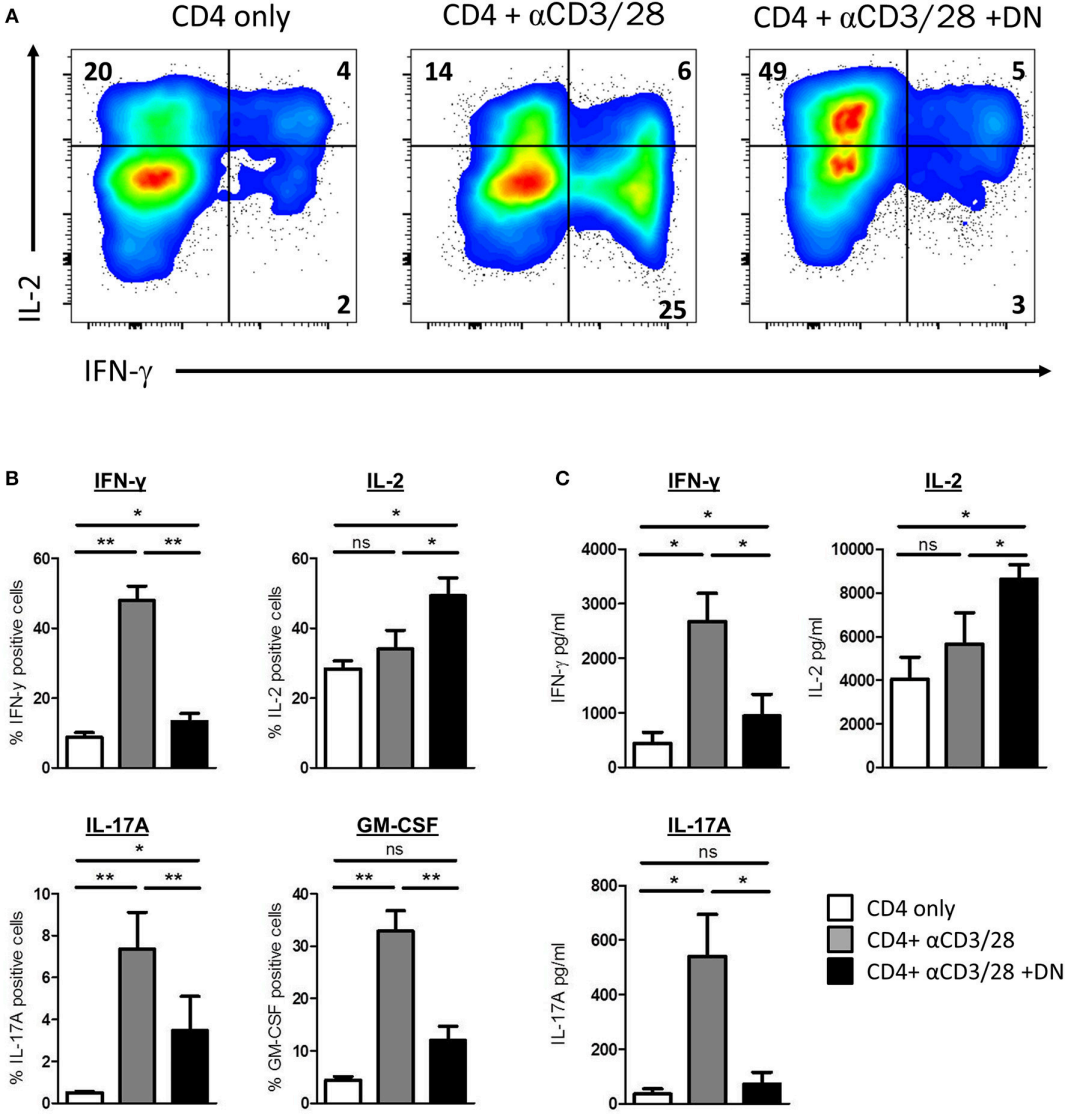
DN T-cells altered cytokine profile of CD4 T-cells. Freshly isolated CD4 T-cells were incubated with anti-CD3/CD28 coated beads in presence or absence of DNT-cells. **(A,B)** On day 6 of co-culture cells were re-stimulated with PMA/Ionomycin in the presence of monensin. Expression of cytokines was determined by intracellular flow cytometry staining. **(A)** Representative dot plots were gated for viable CD4 T-cells. **(B)** Graph represent mean percentages+/– SEM of IFN-γ+, IL-2+, GM-CSF, and IL-17A+ CD4 T-cells of at least seven independent experiments. **(C)** On day 6 of co-culture CD4 T-cells were re-isolated by magnetic sorting and stimulated with PMA/Ionomycin for 6 h. Secretion of effector cytokines in the supernatant was analyzed for of IFN-γ, IL-2, and IL-17A by cytometric bead array. Graphs show concentration +/– SEM of indicated cytokines, data represent results of at least seven independent experiments. ns, not significant, ^*^p < 0.05, ^**^p < 0.01.

We next sought to investigate whether CD4 T-cells offer significant functional changes after co-culture with DN T-cells. Therefore, we assessed cytokine production of CD4 T-cells by flow cytometry. Activated CD4 T-cells produced substantial amounts of IFN-γ (Figures 5A,B). Strikingly, CD4 T-cells activated in presence of DN T-cells did not show enhanced IFN-γ production but more IL-2 expression. Moreover, we observed that the GvHD-associated effector cytokines IL-17A and GM-CSF were also reduced in CD4 T-cells after DN T-cell co-culture (Figure 5B). To confirm these findings, we re-separated CD4 T-cells from DN T-cells and performed cytometric bead arrays. Activation of CD4 T-cells with anti-CD3/CD28 coated beads resulted in an enhanced secretion of cytokines IFN-γ and IL-17A, suggesting a differentiation into CD4 effector T-cells (Figure 5C). In contrast, CD4 T-cells cultured with DN T-cells did not upregulate secretion of effector cytokines after re-stimulation, while IL-2 production was increased compared to unstimulated or activated CD4 T-cells. In summary, our findings provide evidence that DN T-cells inhibit proliferation but also reprogram effector functions of CD4 T-cells.

### Modulation of CD4 T-Cells Is Cell-Cell-Contact Dependent

**Figure 6:**
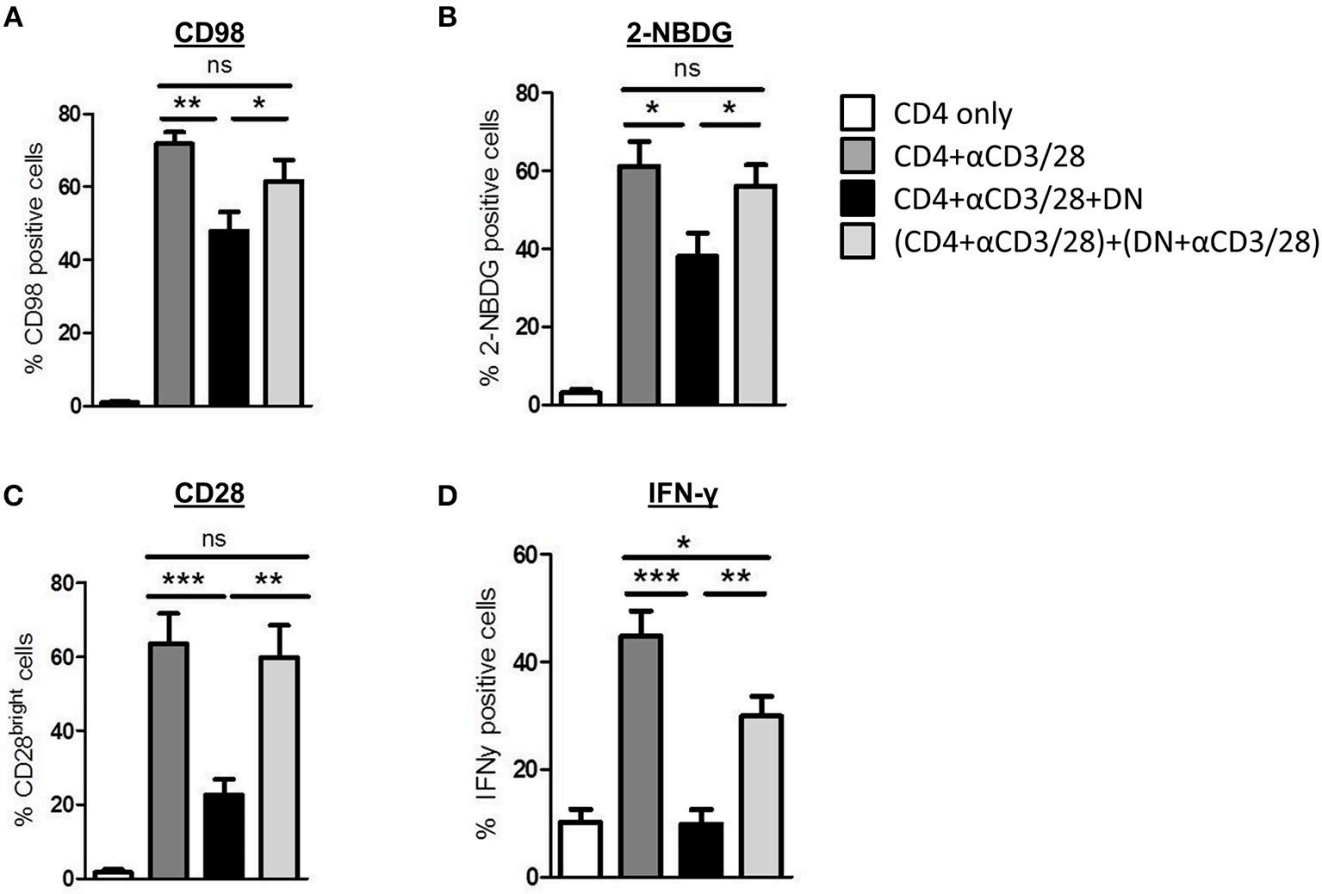
DN T cell-mediated modifications of CD4 T cell phenotype, metabolism, and cytokine production are cell-cell-contact dependent. CD4 T-cells were incubated with anti-CD3/CD28 coated beads, DN T-cells were added directly to the culture or to the top chamber of a transwell system as described in *Materials and Methods*. **(A,B)** Expression of CD98 **(A)** and uptake of 2-NBDG **(B)** was assessed after 3 days, data show mean percentages +/– SEM of at least seven independent experiments. **(C)** Expression of CD28 was assessed after 6 days, data show mean percentages of CD28 bright-cells +/– SEM of seven independent experiments. **(D)** On day 6 of co-culture cells were stimulated with PMA/Ionomycin in the presence of monensin for 4 h. Expression of IFN-γ was determined by intracellular flow cytometry staining. Graphs illustrate mean percentages +/– SEM of IFN-γ on CD4 T-cells, data of seven independent experiments is shown. ns, not significant, ^*^p < 0.05, ^**^p < 0.01, ^***^p < 0.001.

**Figure 7:**
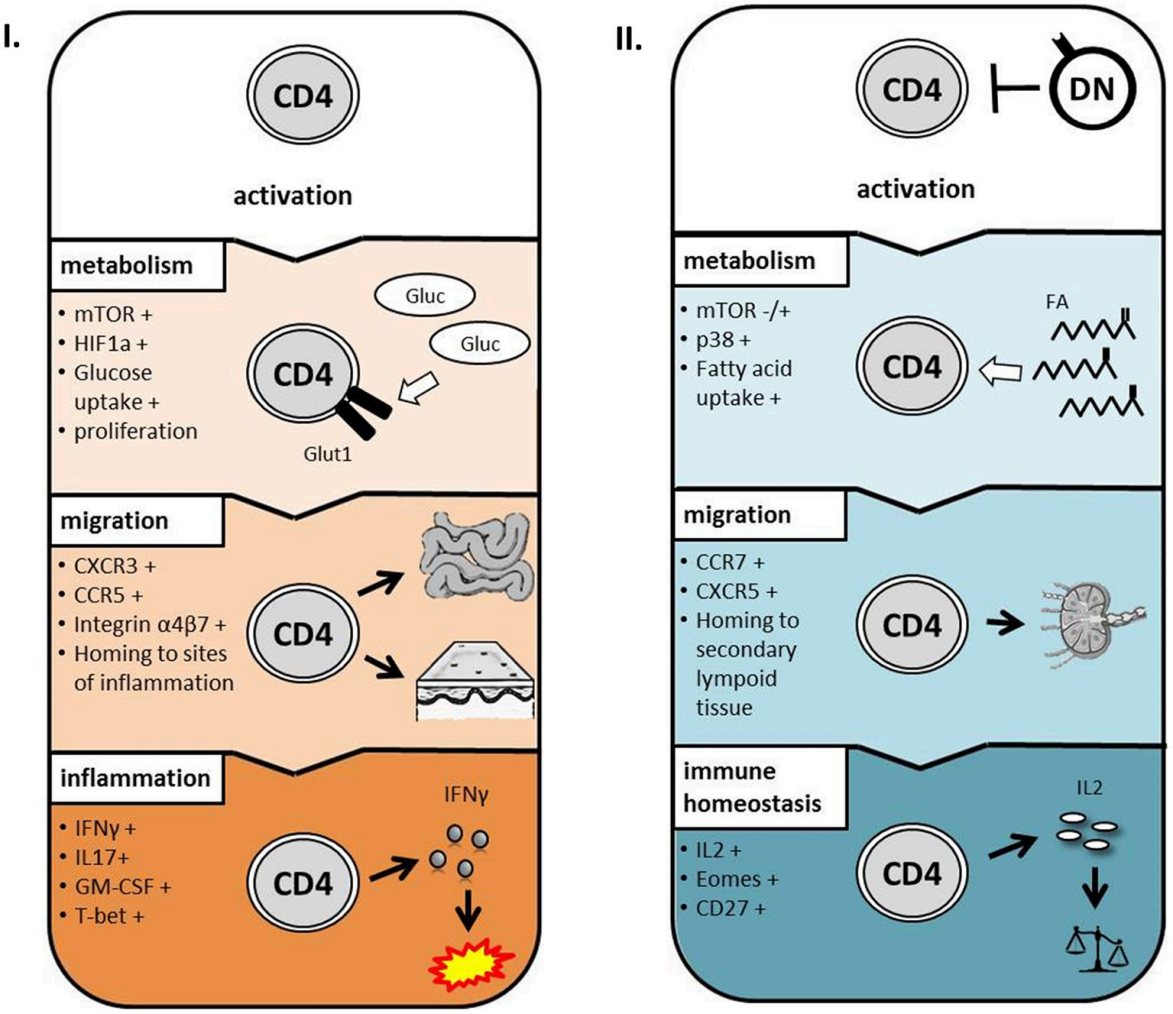
Proposed model for the impact of DN T-cells on CD4 T-cells. After activation, CD4 T-cells upregulate mTOR signaling, glucose metabolism, and chemokine receptors to inflammatory sites resulting in inflammatory conditions **(I.)**. In presence of DN T-cells, metabolism, and migratory capacity of CD4 T-cells are altered during activation resulting in immune homeostasis **(II.)**.

Next we addressed the question whether modulation of CD4 T-cell metabolism and function is induced by cell-cell-contact between DN T-cells and responder CD4 T-cells or results from competition for nutrients or stimulation. First, supernatants obtained from suppression assays were not able to exert any DN T-cell-included alterations when added to freshly with anti-CD3/CD28 coated beads activated CD4 T-cells (data not shown). Moreover, we co-cultured CD4 and DN T-cells in a transwell plate to prevent cell-cell contact but maintain diffusion of nutrients and cytokines. As illustrated in Figures 6A, and B DN T-cells were limited to suppress CD98 expression and glucose uptake in CD4 T-cells when direct cell contact was blocked. Furthermore, CD28 reduction and inhibition of IFN-γ production (Figures 6C,D and data not shown) were reduced in CD4 T-cells after co-cultivation with DN T-cells in the transwell system. These data disclose that cell-cell-contact is indispensable for the alteration of CD4 T-cells by DN T-cells. In summary, these data indicate that human DN T-cells suppress proliferation but also modulate migratory and effector functions of CD4 T-cells via controlling mTOR signaling and metabolic reprogramming (Figure 7).

## Discussion

In this study, we examine the impact of human DN T-cells on CD4 T-cell fate and function and reveal that DN T-cells not only efficiently suppress proliferation of CD4 T-cells (9, 30), but also modulate their metabolic programming and functionality. These findings unveil new mechanisms how DN T-cells can affect induction or maintenance of peripheral tolerance. Notably, DN T-cells have emerged as a promising therapeutic option for a number of entities including GvHD after allo-HSCT. In murine models, DN T-cells were demonstrated to restrict the development of GvHD while mediating beneficial anti-leukemic effects (18). Moreover, the transfer of human DN T-cells in a humanized mouse model was shown to delay onset of xenogeneic GVHD (21). Clinical studies in patients after allo-HSCT revealed an inverse correlation between DN T-cell levels and severity of both acute and chronic GvHD (19, 20), suggesting a therapeutic potential of human DN T-cells.

Using DN T-cells to modulate functionality of T-cells could offer an additional advantage over standard therapy with immunosuppressive drugs in GvHD treatment. The metabolic master regulator mTOR integrates nutrient and energy sensing pathways and controls proliferation, differentiation and metabolism of T-cells. Dysregulation of mTOR signaling has been reported in various autoimmune diseases and transplantation settings and can be targeted by immunosuppressive drugs like sirolimus and everolimus (31, 32). Recent reports have clearly demonstrated that mTOR signaling induce metabolic reprogramming of alloantigen activated T-cells after allo-HSCT. Moreover, the authors have identified glycolysis as the predominant metabolic process used by alloreactive T-cells to promote GvHD (33). Our results showed that DN T-cells can especially inhibit the mTOR pathway in CD4 T-cells but spare other T-cell signaling pathways, as p38 and NFκB were unaffected. Consistent with this observation, we found that DN T-cells downregulate glucose metabolism and uptake in CD4 T-cells, whereas uptake of fatty acids was not affected. In the literature various molecular mechanisms have been described to regulate mTOR signaling and metabolic reprogramming of T-cells as for example the inhibitory molecules CTLA-4 and PD-1 engage distinct phosphatases to terminate mTOR phosphorylation (34, 35). Interestingly, the inhibitory receptor PD-1 has also been reported to block glycolysis but favors fatty acid oxidation in T-cells (36). Further analysis has to be done to determine the underlying mechanism of DN T-cells-mediated metabolic alterations. Anyhow, these findings are of particular interest as selective targeting of metabolic pathways in T-cells offers new opportunities to specifically suppress alloreactive T-cells (37).

Several studies have shown that cell metabolism determines T-cell fate and function. Notably, mTOR signaling and metabolic reprogramming are involved in the differentiation of memory and effector T-cells by regulation of the transcription factors T-bet and Eomes (38). We found that CD4 T-cells revealed elevated levels of Eomes but reduction of T-bet expression after co-culture with DN T-cells. Moreover, these cells displayed an altered phenotype with enhanced expression of CD27, CCR7, and CXCR5, which are described to be discriminatory for long-living central-memory T-cells (39–41). Of interest, Rapamycin-treated T-cells were reported to be dependent on oxidative phosphorylation and more prone to become long-living memory T-cells, suggesting that specific mTOR inhibition induce these phenotypic alterations (42).

Of particular importance was the observation that suppressed CD4 T-cells have different effector functions, namely decreased IFNγ, IL-17A, and GM-CSF levels but amplified IL-2 production. Altered secretion of effector cytokines by CD4 T-cell due to DN T-cells could have important implications for the onset of GvHD in a clinical setting after allo-HSCT. The main Th1 effector cytokine IFN-γ plays an essential role in the induction of GvHD as grafts of IFN-γ-gene knockout donors could not cause GvHD in recipient mice (43) and, in turn, GvHD could not be induced in IFN-γ-signaling deficient mice (44). These defects in cytokine production are in support of the idea that DN T-cells impair glucose metabolism as aerobic glycolysis is fundamental for IFN-γ translation (45). In addition, IL-17A and GM-CSF secretion of CD4 T-cells were diminished by DN T-cells. Both IL-17A serum levels and Th17 infiltrating cells are associated with GvHD after allo-HSCT (46, 47). IL-17A expression is associated with glucose metabolism as the transcription factor HIF-1α controls the activation of the IL-17A promoter and Th17 differentiation (48). Moreover, a recent study has demonstrated GM-CSF producing T-cells to be sufficient to promote GvHD (49). In contrast, our data revealed enhanced IL-2 production by CD4 T-cells after co-culture with DN T-cells. IL-2 is required for T-cell activation, differentiation, and survival but can also be favorable as IL-2 selectively can restore the immunosuppressive function of FoxP3 Tregs without activation of T-cells or abrogation of anti-leukemia effects (50). In addition to cytokines, chemokines also play a crucial role in T-cell effector function. Indeed, the pro-inflammatory chemokine receptors CXCR3 and CCR5 are involved in the induction of GvHD by orchestrating the migration and infiltration of effector T-cells to their target tissue (28, 29). Moreover, the integrin α4β7 is exclusively responsible for alloreactive T-cells homing to the gut (51). Of note, an antagonist of CCR5 (Maraviroc) or therapeutic antibodies against α4β7 (Vedolizumab, Natalizumab) are currently examined in clinical trials and raise hopes for a novel therapy to treat GvHD (52, 53). Our findings suggest that DN T-cells also have potential to shift the expression of chemokine receptors on CD4 T-cells and thus induce homing to secondary lymphoid organs rather than sites of inflammation. Since coordinated migration of cells by chemokine receptors is required for the appropriate execution of T-cell effector function, DN T-cells open up another possibility to interfere with T-cell function after allo-HSCT.

In summary, T-cells do not maintain their naïve phenotype after DN T-cell co-culture, but display characteristics akin to long-living central-memory T-cells. Our observations shed light on the molecular process of DN T-cell-mediated suppression, since the T-cells are not rendered senescent or anergic. This is in contrast with recent studies that have shown FoxP3+ Tregs to induce senescence in T-cells and FoxP3+ Treg-treated T-cells do not exhibit a similar modulation of surface molecules or cytokine production (54). Furthermore, cellular-based therapy after HSCT with FoxP3+ Tregs has been already tested. Initial clinical studies obtained first incidence that adoptive transfer of *ex vivo* expanded FoP3+ Tregs can prevent GvHD (6, 7). DN T-cells are another promising regulatory T-cell subset, which not only abolishes but modulates target cell function. In addition, DN T-cells also might be able to support FoxP3+ Tregs after adoptive transfer by enhancing IL-2 production of T-cells.

In conclusion, our results reveal new and various targets of DN T-cells to selectively modulate signaling and metabolic programming of T-cells resulting in functional altered effector cells. These findings could pave the way to use DN T-cells for cellular therapy as an alternative treatment strategy to prevent and diminish GvHD after allo-HSCT.

## Ethics Statement

This study was carried out in accordance with the recommendations of Ethics committee of the University Erlangen-Nuremberg with written informed consent from all subjects. All subjects gave written informed consent in accordance with the Declaration of Helsinki. The protocol was approved by the Ethics committee of the University Erlangen-Nuremberg (protocol number 284-18 Bc).

## Author Contributions

TH, AM, and SV designed the research. TH, MA, MP, CS, and SV performed experiments. TH, MA, KH, DM, HB, AM, and SV analyzed and interpreted data. TH, AM, and SV wrote the manuscript. HB, DM, and AM provided advice and revised the manuscript. All of the authors edited the manuscript.

### Conflict of Interest Statement

The authors declare that the research was conducted in the absence of any commercial or financial relationships that could be construed as a potential conflict of interest.
