# Supplementary material for: Human Double-Negative Regulatory T-Cells Induce a Metabolic and Functional Switch in Effector T-Cells by Suppressing mTOR Activity
Source: Front Immunol. 2019 Apr 26;10:883. doi: 10.3389/fimmu.2019.00883 (PMC6498403; doi:10.3389/fimmu.2019.00883)

Supplemental Figure 1. DN T-cells inhibit mTOR activation but not MAPK p38 signaling in CD4 T-cells. Freshly isolated CD4 T-cells were incubated with anti-CD3/CD28 coated beads in absence (grey) or presence (black) of DNT-cells. Unstimulated CD4 T-cells were used as negative control (white). Representative histogram of one experiment is shown. MFI is indicated. (A) Phosphorylation of ribosomal protein S6(S240) (left) and MAPK p38(T180/Y182) (right) in CD4 T-cells after 24h culture was quantified by flow cytometry. (B) On day 1 of co-culture CD4 T-cells were re-isolated by magnetic sorting and fixed on a 96-well flat bottom plate. Expression of total-Akt, phospho-Akt, total-p38 and phospho-p38 was assessed with ELISA in activated CD4 T-cells in absence (grey) or presence (black) of DN T-cells. Negative control is not treated with a primary Antibody (white). ns not significant, *p<.05, ***p<.001. (C) Expression of HIF1α and NFκB(p65) was analyzed in CD4 T-cells after 24h co-culture. (D) Expression of CD98 and CD54 was measured after 3 days.

Supplemental Figure 2. DN T-cells impair metabolic reprogramming of CD4 T-cells. Freshly isolated CD4 T-cells were incubated with anti-CD3/CD28 coated beads in absence (grey) or presence (black) of DN T-cells, unstimulated CD4 T-cells served as negative control (white). Cells were analyzed by flow cytometry after 3 and 6 days. Representative histogram of one experiment is shown. MFI is indicated. (A) Expression of GLUT1 and GLUT3 in CD4 T-cells was determined by flow cytometry after 3 and 6 days. (B) CD4 T-cells were incubated with 20 µM MHY-1485 (mTOR++) for 2 hours at 37 °C and washed intensively. Treated and untreated CD4 T-cells were stimulated with anti-CD3/CD28 coated beads in presence or absence of DNT-cells for 3 days. Cells were analyzed for GLUT1 by flow cytometry, histograms were gated for CD4 T-cells. (C) Uptake of the glucose analogue 2-NBDG and the fatty acid Bodipy$_{C1-C12}$in CD 4 T-cells was measured as described in Materials and Methods.

Supplemental Figure 3. DN T-cells modulate expression profiles of CD4 T-cells. Freshly isolated CD4 T-cells were cultured with anti-CD3/CD28 coated beads in absence (grey) or presence (black) of DN T-cells, unstimulated CD4 T-cells were used as negative control (white). Cells were harvested on day 6 of co-culture and analyzed by flow cytometry. Representative histogram of one experiment is shown. MFI is indicated. (A) CD4 T-cells were analyzed for the expression of transcription factors T-bet and Eomes. (B) Expression of CD28 and CD27 on CD4 T-cells is shown. (C) Freshly isolated CD4 T-cells were incubated with 20 µM MHY-1485 (mTOR++) for 2 hours at 37 °C and washed intensively. Treated and untreated CD4 T-cells were stimulated with anti-CD3/CD28 coated beads in presence or absence of DNT-cells for 3 days. Cells were analyzed for CD28 and CD98 by flow cytometry, histograms were gated for CD4 T-cells. (D) CD4 T-cells were analyzed for the expression of transcription factor FoxP3. Data of 10 independent experiments is shown. ns not significant, *p<.05, **p<.01. (E) Representative dot plots for CCR7 and CD45RO were gated for viable CD4 T-cells.

Supplemental Figure 4. DN T-cells influence migratory capacity of CD4 T-cells. CD4 T-cells were incubated with anti-CD3/CD28 coated beads absence (grey) or presence (black) of DN T-cells, unstimulated CD4 T-cells were used as negative control (white). After 6 days, cells were analyzed by flow cytometry. Representative histogram of one experiment is shown. MFI is indicated. Data represent expression of (A) CXCR3, CCR5 and (B) CCR7 and CXCR5 on CD4 T-cells. (C) Freshly isolated CD4 T-cells were incubated with 20 µM MHY-1485 (mTOR++) for 2 hours at 37 °C and washed intensively. Treated and untreated CD4 T-cells were stimulated with anti-CD3/CD28 coated beads in presence or absence of DNT-cells for 3 days. Cells were analyzed for CXCR3 by flow cytometry, histograms were gated for CD4 T-cells.

Supplemental Fig. 1

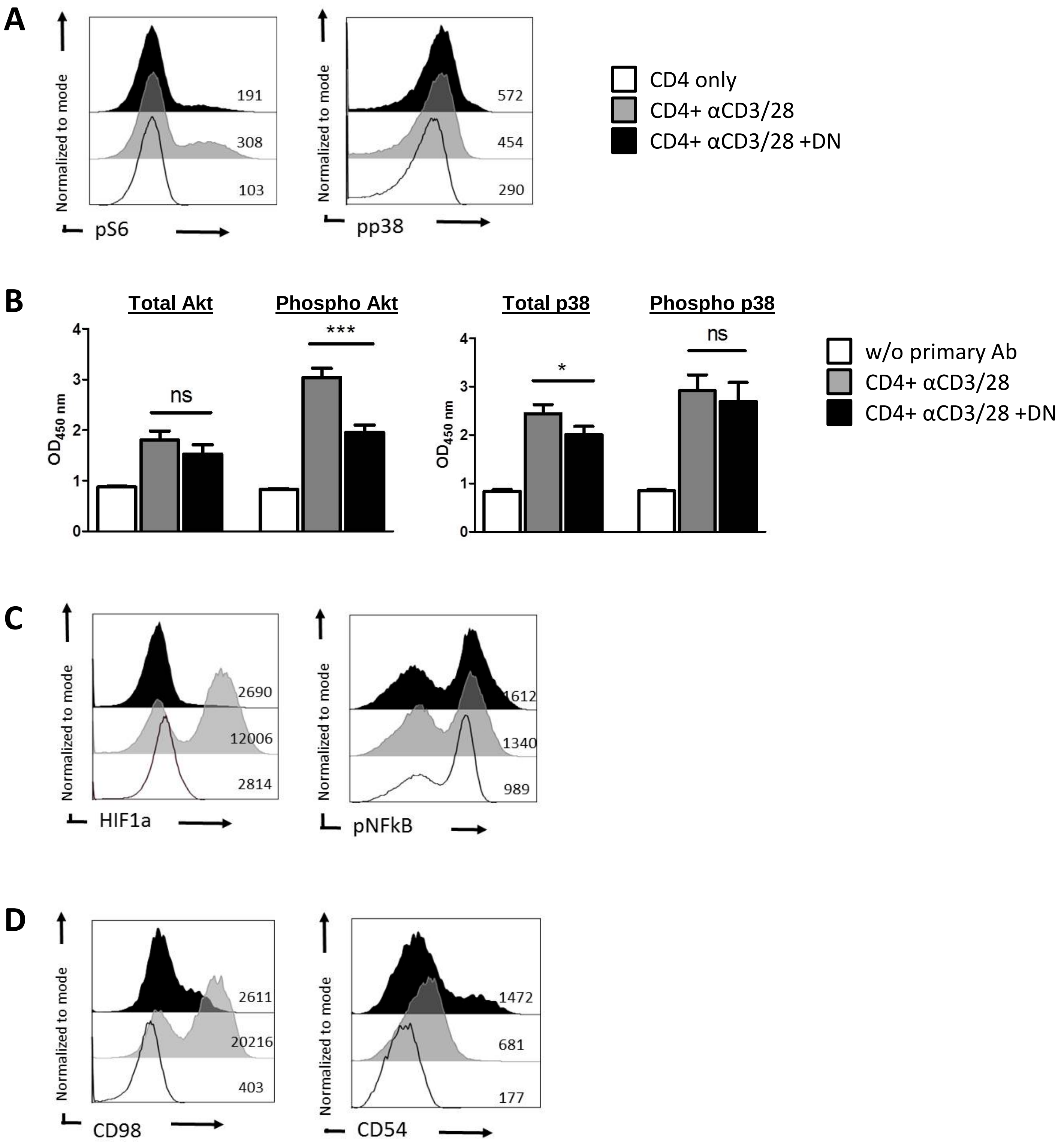

Supplemental Fig. 2

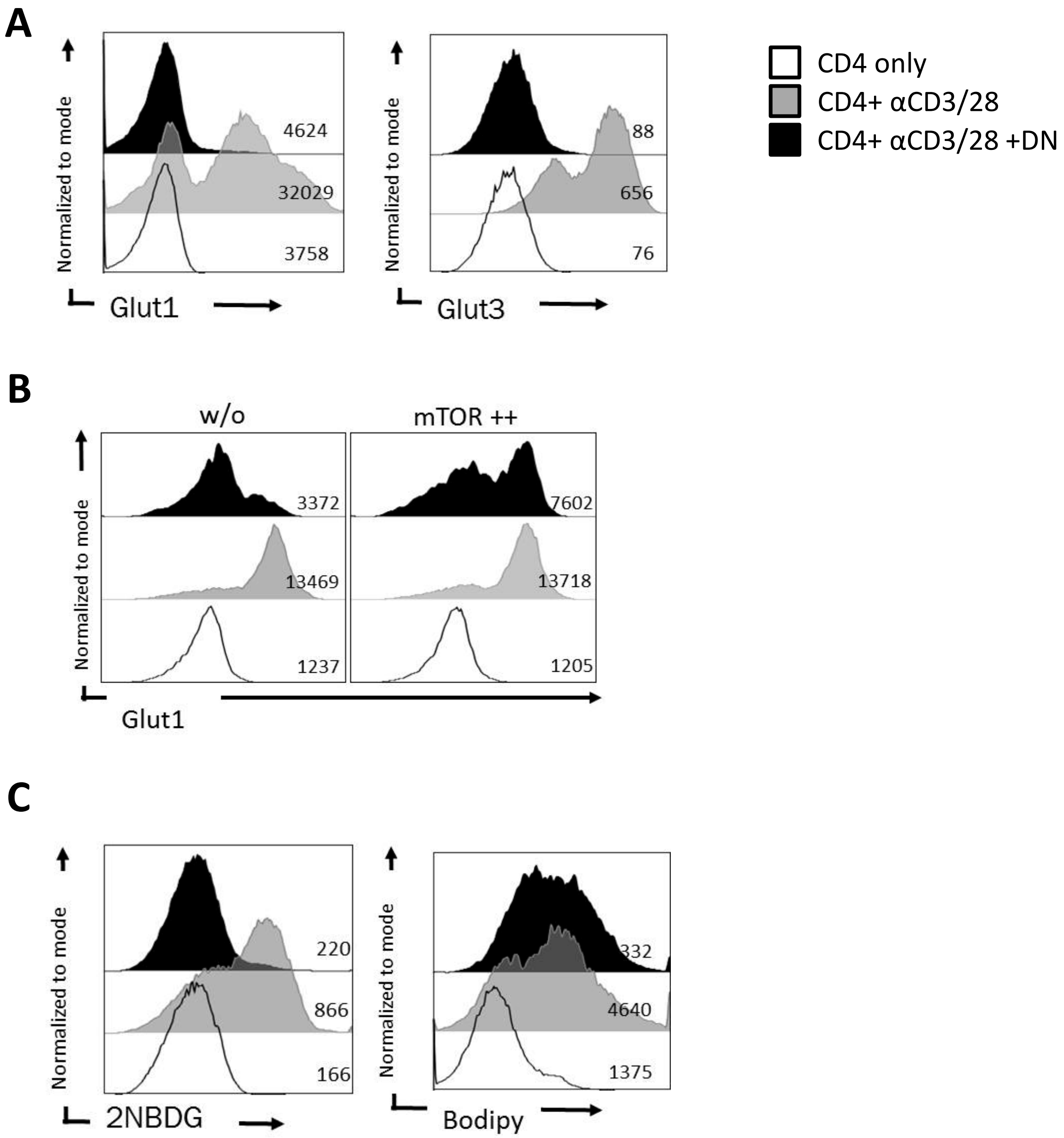

Supplemental Fig. 3

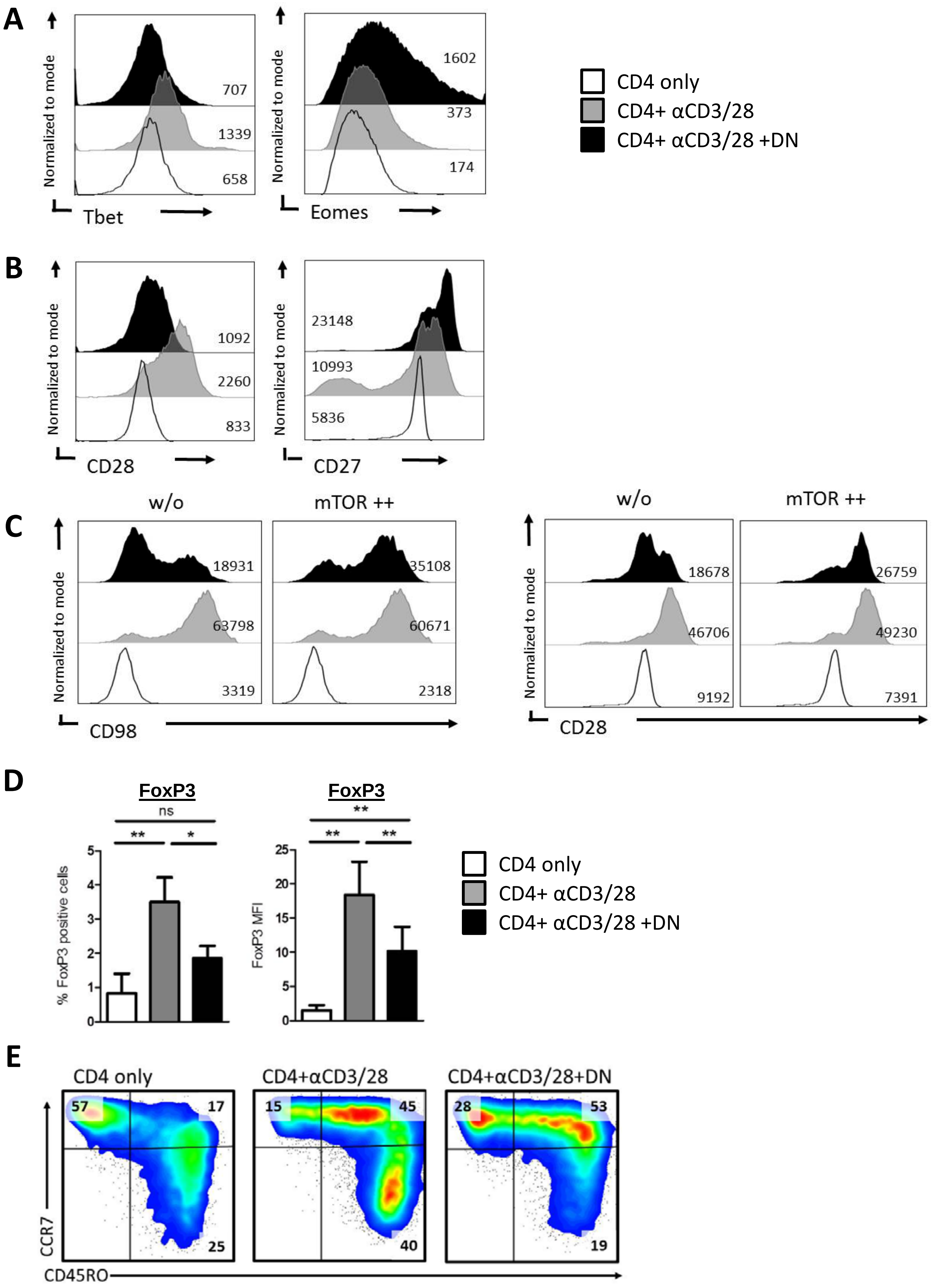

Supplemental Fig. 4

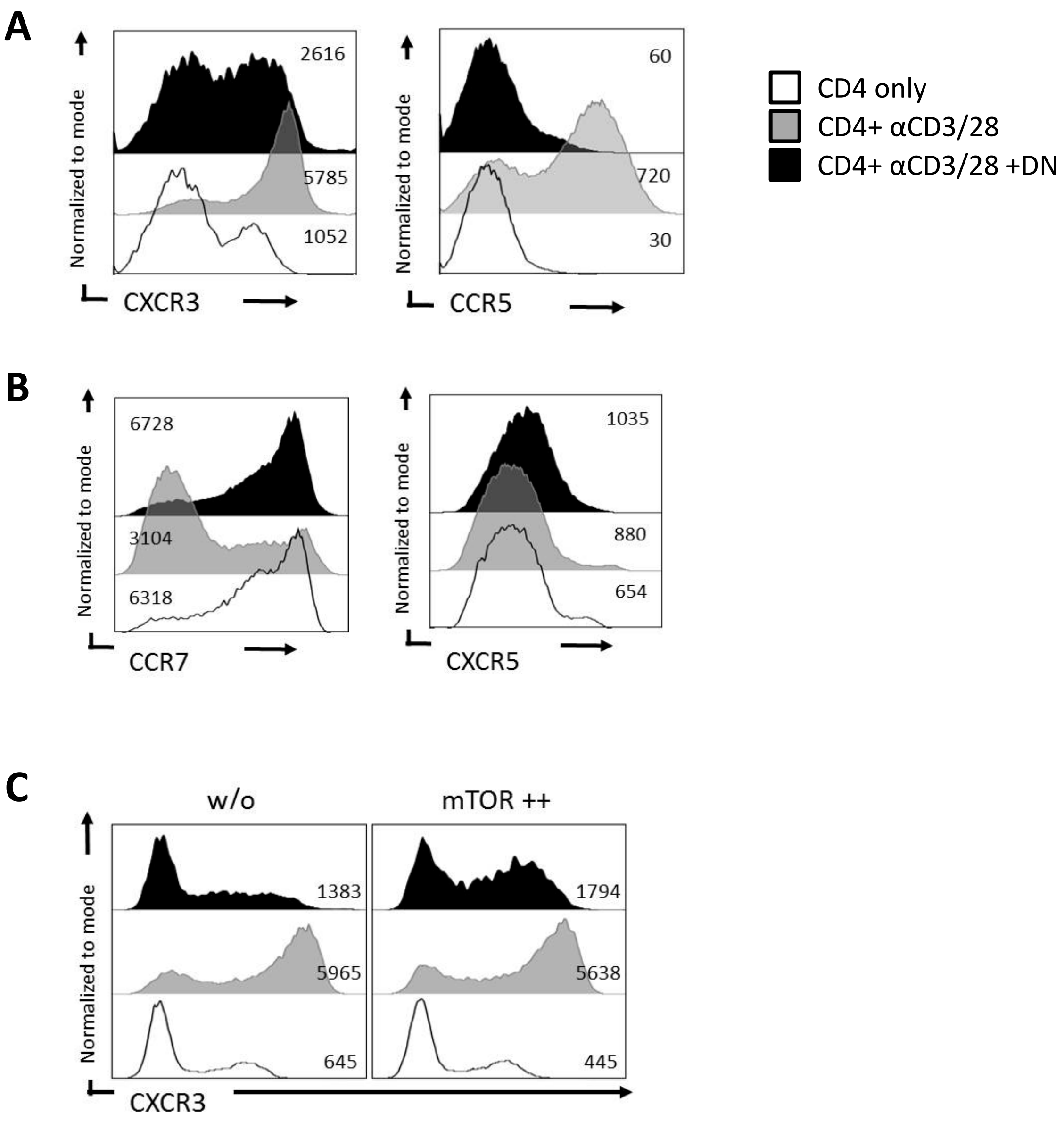

Supplement: Supplementary file 1 [file Data_Sheet_1.PDF]
